# Editorial: TGF-**β** and BMP signaling in cancer

**DOI:** 10.3389/fcell.2022.1012326

**Published:** 2022-09-02

**Authors:** Xiaohua Yan, Long Zhang, Keiji Miyazawa, Peter ten Dijke

**Affiliations:** ^1^ Department of Neurosurgery, The Second Affiliated Hospital of Nanchang University, Nanchang, China; ^2^ Department of Biochemistry and Molecular Biology, School of Basic Medical Sciences, Nanchang University, Nanchang, China; ^3^ MOE Laboratory of Biosystems Homeostasis and Protection and Innovation Center for Cell Signaling Network, Life Sciences Institute, Zhejiang University, Hangzhou, China; ^4^ Department of Biochemistry, Graduate School of Medicine, University of Yamanashi, Yamanashi, Japan; ^5^ Öncode Institute and Department Cell and Chemical Biology, Leiden University Medical Centre, Leiden, Netherlands

**Keywords:** bone morphogenetic protein, epithelial-to-mesenchymal transition, tumor microenvironment, SMAD, transforming growth factor-β

Transforming growth factor-β (TGF-β) family members, which includes TGF-βs, activins and bone morphogenetic proteins (BMPs) are structurally related secreted cytokines that fulfil key roles during embryonic development and in maintaining tissue homeostasis (Siegel and Massagué, 2003). Perturbation of these cytokine actions may lead to various diseases, including cancer. TGF-β family members signal via specific transmembrane type I and type II serine/threonine kinase receptors and intracellular SMAD transcriptional effector proteins. The type I receptors are phosphorylated by type II kinases and determine the signaling specificity within the cell surface ligand-receptor complex. Whereas type I receptors for TGF-β and activin (i.e., TβRI or activin receptor-like (ALK)5 and ActR-IB or ALK4, respectively) signal via SMAD2 and SMAD3, two receptor-regulated SMADs (R-SMADs), BMP type I receptors (ALK1, ALK2, BMPRIA or ALK3, and BMPRIB or ALK6) activate three R-SMADs, i.e. SMAD1, -5 and -8 by direct phosphorylation at the C-terminus. Activated R-SMADs form complexes with the common mediator SMAD4, and then the Smad complexes accumulate within the nucleus. There they act in concert with transcriptional co-activators/co-repressors and epigenic regulators to regulate specific gene transcriptional responses. Next to the canonical SMAD pathway, TGF-β family receptors can also signal via non-SMAD signaling pathways (Heldin and Moustakas, 2016).

TGF-β family members are multifunctional cytokines and elicit effects that are highly dependent on cellular context (Morikawa et al., 2016; David and Massagué, 2018). In cancer, TGF-β family members have been attributed with both tumor suppressive and tumor promoting activities. Among all its family members, the action of TGF-β in cancer has been investigated the most. It is likely that many of the observations for TGF-β also apply (with some variation) to other family members. In normal cells, pre-malignant and even some malignant tumor cells, TGF-β restrains cell proliferation, induces apoptosis and contributes to genome stability. Cancer cells can become insensitive to these tumor suppressive effects when receptors or SMADs become mutated or dysfunctional in other ways. As a result, these cells may undergo uncontrolled growth. Moreover, in advanced cancer cells, when proto-oncogenes and tumor suppressor genes have become activated or inactivated, respectively, cells not only become insensitive to the TGF-β-induced cytostatic and pro-apoptotic effects, but may also use the SMAD pathway to stimulate pro-oncogenic effects, such as induction of the epithelial-to-mesenchymal transition (EMT) programme and thereby promote cancer cell invasion and metastasis (Katsuno et al., 2013). Moreover, besides cancer cells, host cells can secrete high amounts of TGF-β, which acts not only on cancer cells but also cells from the tumor microenvironment, thereby stimulating tumor angiogenesis and immune evasion (Battle and Massague, 2019; Liu et al., 2021).

While targeting TGF-β signaling by interfering with TGF-β-receptor interaction or inhibiting the receptor kinase activity for cancer therapy has been pursued by many academic and company laboratories, still no TGF-β inhibitor has been clinically approved. Part of this can be attributed to the fact that the inhibitors tested in clinical trials do not act in a cell type specific manner, and when administered systemically lead to on target toxic side effects. Recently, however, we see a renewed interest in targeting this pathway by (selectively) interfering with the TGF-β-induced immune suppression as it may allow for more effective immune checkpoint inhibitor therapy (Battle and Massagué, 2019; Liu et al., 2021).

This Research Topic comprises five original research and six review (including one minireview) articles covering diverse and complementary aspects on the role of TGF-β and BMPs in cancer progression. Shuelten and Zhang provide an overview on the important role of TGF-β in the tumor microenvironment. In particular, they focus on how TGF-β acts as a potent differentiation factor for epithelial and endothelial cells and fibroblasts, a polarizing agent for macrophages and a mediator of metabolic changes, which all contribute to epithelial tumor formation. How the interplay between TGF-β signaling and metabolism controls cellular homeostasis and contributes to cancer progression is discussed in depth by Liu and Chen. A picture is emerging that many metabolic pathways are controlled in a highly cell type-dependent manner. Zhang et al. focus in their review on involvement of TGF-β as a pivotal driver of cancer therapy resistance. Combining TGF-β inhibitors with immune therapies has promise but will require careful selection of patients that benefit most from treatment. Extracellular vesicles (EVs) are emerging as an important mechanism for cell communication. Multiple TGF-β signaling components have been shown to be present within or associated with these vesicles, and were reported to play a pivotal role in cancer metastasis, immune evasion and therapy resistance. In addition, the TGF-β signaling components that are associated with EVs may have potential as biomarkers for prognosis, diagnosis and therapy prediction (Rodrigues-Junior et al.). Whereas the preceding papers mainly focused on TGF-β, Ehata and Miyazono and Guyot et al. present their reviews the roles of BMPs in cancer progression. Like TGF-β, BMPs have both pro-tumorigenic and tumor suppressive roles. Ehata and Miyazono discuss the application of BMP signaling inhibitors for cancer treatment. Guyot et al. focus on the parallel actions of BMP2/4 in myeloid leukemia and breast cancer.

In an original research paper, Shi et al. report on how TGF-β enriched CAFs act as a critical determinant for lung metastasis of squamous cell carcinoma (SCC). They propose that these cells may provide a prognostic marker and therapeutic target for metastasis of SCC to lungs. Trelford and Di Guglielmo delineate the critical involvement of SMAD4 and the TAK1-TRAF6-p38 MAPK pathway in TGF-β-induced autophagy in non-small cell lung cancer cells (NSCLCs). Using publicly available databases, Tu et al. analyse the potential role of SNAI family members in breast cancer prognosis and immune regulation. Using a similar bioinformatic approach, Gao and Zhou analyze mRNA expression levels of RUNX family members and observe their correlations with prognosis and immune cell infiltration in breast cancer. Huang et al. ascribe in their original research article a key effector role for a member of the Paraneoplastic Ma family, i.e. PNMA5, as a downstream BMP2 effector in mediating the bone metastasis of NSCLCs. It will be interesting to explore the therapeutic targeting of PNMA5 for the treatment of NSCLC bone metastasis.

Taken together, the current Research Topic provides valuable new insights into the role of TGF-β and BMPs in cancer progression. New directions of future research are offered on how to further explore the multifaceted role that TGF-β members play therein. We anticipate that soon the efforts in fundamental and translational research will lead to the clinical approval of a drug that targets a TGF-β family member for the treatment of specific cancer subtypes.
